# Homocysteine Metabolism Gene Polymorphisms (MTHFR C677T, MTHFR A1298C, MTR A2756G and MTRR A66G) Jointly Elevate the Risk of Folate Deficiency

**DOI:** 10.3390/nu7085303

**Published:** 2015-08-10

**Authors:** Wen-Xing Li, Shao-Xing Dai, Jun-Juan Zheng, Jia-Qian Liu, Jing-Fei Huang

**Affiliations:** 1 Institute of Health Sciences, Anhui University, Hefei 230601, Anhui, China; E-Mail: liwenxin@mail.kiz.ac.cn; 2 State Key Laboratory of Genetic Resources and Evolution, Kunming Institute of Zoology, Chinese Academy of Sciences, Kunming 650223, Yunnan, China; E-Mails: daishaoxing@mail.kiz.ac.cn (S.-X.D.); zhengjunjuan@mail.kiz.ac.cn (J.-J.Z.); liujiaqian@mail.kiz.ac.cn (J.-Q.L.); 3 KIZ-SU Joint Laboratory of Animal Models and Drug Development, College of Pharmaceutical Sciences, Soochow University, Kunming 650223, Yunnan, China; 4 Collaborative Innovation Center for Natural Products and Biological Drugs of Yunnan, Kunming 650223, Yunnan, China

**Keywords:** *MTHFR* C677T, *MTHFR* A1298C, *MTR* A2756G, *MTRR* A66G, folate deficiency

## Abstract

Folate deficiency is strongly associated with cardiovascular disease. We aimed to explore the joint effect of the methylenetetrahydrofolate reductase (MTHFR) C677T and A1298C, methionine synthase (MTR) A2756G, and methionine synthase reductase (MTRR) A66G polymorphisms on folate deficiency in a Chinese hypertensive population. A total of 480 subjects aged 28–75 were enrolled in this study from September 2005–December 2005 from six hospitals in different Chinese regions. Known genotypes were detected by PCR-RFLP methods and serum folate was measured by chemiluminescence immunoassay. Our results showed that *MTHFR* 677TT and *MTR* 2756AG + GG were independently associated with a higher risk of folate deficiency (TT *vs.* CC + CT, *p* < 0.001 and AG + GG *vs.* AA *p* = 0.030, respectively). However, the *MTHFR* A1298C mutation may confer protection by elevating the serum folate level (*p* = 0.025). Furthermore, patients carrying two or more risk genotypes showed higher odds of folate deficiency than null risk genotype carriers, especially those carrying four risk genotypes. These findings were verified by generalized multifactor dimensionality reduction (*p* = 0.0107) and a cumulative effects model (*p* = 0.001). The results of this study have shown that interactions among homocysteine metabolism gene polymorphisms lead to dramatic elevations in the folate deficiency risk.

## 1. Introduction

Recently, a large-scale randomized clinical trial confirmed the benefits of folate therapy on the risk of first stroke [1]. A previous meta-analysis has shown that the effect of the *MTHFR* C677T variant on the homocysteine concentration is modified by folate status [2]. Hyperhomocysteinemia and the *MTHFR* 677TT genotype are considered risk factors for cardiovascular diseases (CVDs) [2,3,4,5]. The associations between homocysteine metabolism gene polymorphisms and homocysteine, folate, and other B vitamins have been widely studied [6]. Some mutations may result in an elevation in the plasma homocysteine concentration and a reduction in the folate concentration [6,7,8,9,10,11], thereby exacerbating the risks of several complicated diseases [6,12]. Other studies have demonstrated that high-dose folate intervention therapy [13] or dietary folate supplementation [8] may increase the serum folate level and simultaneously reduce the prevalence of hyperhomocysteinemia and hypertension.

Folate is a crucial vitamin in homocysteine metabolism. Serum folate enters into tissue cells via folate receptors, and then dihydrofolate reductase (DHFR) converts it into tetrahydrofolate. Next, tetrahydrofolate is transformed into 5, 10-methylenetetrahydrofolate, with vitamin B_6_ as a cofactor [14]. Then, methylenetetrahydrofolate reductase (MTHFR) converts 5, 10-methylenetetrahydrofolate into 5-methyltetrahydrofolate, providing a methyl group for conversion of homocysteine into methionine in a reaction catalyzed by methionine synthase (MTR) [15,16]. MTR requires vitamin B_12_ (cobalamin) as a coenzyme. Over time, the cobalamin (I) cofactor of MTR is oxidized to form cobalamin (II), leading to inactivation of MTR. Thus, methionine synthase reductase (MTRR) is required for reversion of oxidized cobalamin (II) to CH_3_-cobalamin (III) to maintain the activity of MTR [17].

Some common polymorphisms (*MTHFR* C677T, rs1801133; *MTHFR* A1298C, rs1801131; *MTR* A2756G, rs1805087; and *MTRR* A66G, rs1801394) may influence the serum folate level [6,7,10,18]. Numerous studies have demonstrated that the *MTHFR* C677T mutation significantly lowers the serum folate level [7,10,11,19], whereas a recent study has reported no such correlation [20]. The associations of *MTHFR* A1298C and *MTR* A2767G with the folate level remain controversial [10,18]. In addition, the *MTRR* A66G polymorphism itself may not affect the plasma folate level [7]. Further, these mutations may synergistically promote folate deficiency [21,22]. A low folate level may increase the risk of hyperhomocysteinemia, as has been demonstrated in 77% of hypertensive patients in a previous study [23]. In many developed countries (USA, Canada, UK, France, and other western countries), folic acid fortification has been fully implemented. This measure has been reported to reduce the risk of complex diseases [2,6,8]. However, no folate fortification policy has been established in China, India, Pakistan or other Asian countries and, thus, folate deficiency is more common in Asian populations than in European and American populations [9,10,11].

The aims of our study were to investigate the associations between homocysteine metabolism gene polymorphisms (*MTHFR* C677T, *MTHFR* A1298C, *MTR* A2756G and *MTRR* A66G) and the serum folate level, as well as to explore the independent and interactive effects of the risk genotypes on the incidence of folate deficiency in the Chinese hypertensive population.

## 2. Experimental Section 

### 2.1. Participants and Procedures

This study was conducted using data collected in a previous study [24]. This was a multicenter, randomized, double-blind controlled trial in hypertensive Chinese adults (clinicaltrials.gov; identifier: NCT00520247). Details regarding “Study subjects”, “Randomization and double blinding”, “Data collection procedures”, and “Laboratory tests” have been previously described [24]. In total, 480 patients with mild or moderate hypertension were recruited from six hospitals in different Chinese regions (Ha’rbin, Shanghai, Shenyang, Beijing, Xi’an, and Nanjing) from September to December 2005. All six hospitals have been certified as clinical pharmacology centers by the State Food and Drug Administration of China. Demographic and clinical information and serum folate and homocysteine concentrations were obtained at baseline. This study was approved by the Ethics Committee of Peking University First Hospital, Beijing, China. The purpose and procedures of the study were carefully explained to all participants, and written informed consent was obtained from each participant.

### 2.2. DNA Extraction and Genotyping

All participants were requested to provide 2 mL peripheral whole blood, which was collected in ethylenediaminetetraacetic acid (EDTA) and stored at −20 °C. DNA was extracted by conventional methods. The TaqMan probe technique was used for detecting polymorphisms in Hcy pathway genes at our central laboratory. The polymerase chain reaction-restriction fragment length polymorphism (PCR-RFLP) method was applied to detect the *MTHFR* C677T, *MTHFR* A1298C, *MTR* A2756G, and *MTRR* A66G genotypes. Each genotyping reaction mixture contained 4 ng dried DNA, 0.08 mL 40 assay locus-specific probe, and 2.0 mL TaqMan universal polymerase chain reaction (PCR) master mix in a final volume of 4 mL, with addition of 1.92 mL sterile water. The main parameters for PCR-RFLP of the four single nucleotide polymorphisms (SNPs) are shown in Table 1. The amplified PCR products were separated on a 3% agarose gel. To ensure the accuracy of genotyping, genotyping calls were observed by two independent researchers. The genotyping call rate for assessments of all genetic variants was ≥98% in this study.

**Table 1:** Primer sequences and reaction conditions for PCR-RFLP of gene polymorphisms.

Gene	Primer sequence ^1^	T ^2^ and Cycles	Product Size	Restriction Enzyme
*MTHFR* C677T	F: 5′-TGAAGGAGAAGGTGTCTGCGGGA-3′	58 °C, 35	198bp	*Hinf* I
	R: 5′-AGGACGGTGCGGTGAGAGTG-3′			
*MTHFR* A1298C	F: 5′-CTTTGGGGAGCTGAAGGACTACTAC-3′	52 °C, 38	163bp	*Mbo* II
	R: 5′-CACTTTGTGACCATTCCGGTTTG-3′			
*MTR* A2756G	F: 5′-GAACTAGAAGACAGAAATTCTCTA-3′	53 °C, 36	189bp	*Hae* III
	R: 5′-CATGGAAGAATATCAAGATATTAGA-3′			
*MTRR* A66G	F: 5′-GCAAAGGCCATCGCAGAAGACAT-3′	60 °C, 35	151bp	*Nsp* I
	R: 5′-GTGAAGATCTGCAGAAAATCCATGTA-3′			

^1^ F: forward primer; R: reverse primer. ^2^ T: annealing temperature.

### 2.3. Statistical Analysis

Statistical analyses were conducting using IBM SPSS software package (version 19.0 for windows; IBM, Inc., Armonk, NY, USA). The results for the categorical variables *(i.e.*, the clinical centers and genotypes) are presented as numbers and percentages of cases. The continuous variables (*i.e.*, age, height, weight, body mass index (BMI), systolic blood pressure (SBP), and diastolic blood pressure (DBP)) are presented as the mean ± standard deviation. The means for the continuous variables in the two groups were compared using t tests, and the prevalences of the categorical variables were compared using χ^2^ tests. Since the serum folate and homocysteine levels were not normally distributed, the geometric means and quartiles were displayed and were analyzed with the Mann-Whitney U test. Hardy-Weinberg equilibrium was also assessed for the genotypic frequencies of the different genes with the χ^2^ test.

The unitary linear regression model was used to assess the associations of the *MTHFR* C677T, *MTHFR* A1298C, *MTR* A2756G, and *MTRR* A66G gene polymorphisms with the logarithmic transformed folate level. Unconditional logistic regression (ULR) was performed to estimate the independent and joint effects of the genotypes on folate deficiency, defined as a serum folate level of less than 10 nmol/L [25]. The trend test with the general linear model (GLM) was used to verify the above results. A two-sided *p* value of <0.05 was considered significant.

Generalized multifactor dimensionality reduction (GMDR, version 0.9, obtained from http://www.ssg.uab.edu/gmdr/) was applied to analyze high-order gene-gene interactions. Some parameters, such as training balance accuracy, testing balance accuracy, *p* value, and cross-validation consistency (CVC), were obtained. The model with the maximum CVC score, the best prediction accuracy, and a *p* value of 0.05 or lower was considered the best model. All analyses were adjusted for potential confounding factors, including sex, age, clinical center, height, and weight.

## 3. Results

A total of 480 patients were recruited for this study. After exclusion of 12 subjects who lacked data on *MTHFR* A1298C (five patients), *MTR* A2756G (six patients) or folate (one patient), a total of 468 subjects were included in our final analysis. Four polymorphisms (*MTHFR* C677T, *MTHFR* A1298C, *MTR* A2756G and *MTRR* A66G) in this population showed no deviation in genotype distribution from expected Hardy-Weinberg equilibrium (*p* values of 0.711, 0.380, 0.862 and 0.393, respectively). Since only four subjects had the 2756GG genotype, the 2756AG and 2756GG genotypes were combined for the following statistical analyses.

### 3.1. Demographic and Clinical Characteristics

There were no significant differences in age, BMI, SBP or the frequencies of the four gene polymorphisms between the males and females (Table 2). However, the females had a higher serum folate level (*p* < 0.001), lower serum homocysteine level (*p* < 0.001), and lower DBP (*p* < 0.001) compared with the males.

**Table 2:** Clinical and epidemiologic characteristics of the population grouped by sex.

Characteristic	Sex		*p*
	Females (*n* = 268)	Males (*n* = 200)	
Age, year	56.7 ± 9.4	56.7 ± 10.7	0.990
Height, cm	157.6 ± 5.3	169.6 ± 5.9	<0.001
Weight, kg	64.4 ± 9.5	73.3 ± 10.6	<0.001
Body mass index, kg/m^2^	25.9 ± 3.6	25.4 ± 3.1	0.115
Systolic blood pressure, mm Hg	154.6 ± 12.0	153.8 ± 11.5	0.465
Diastolic blood pressure, mm Hg	92.0 ± 8.2	95.0 ± 8.7	<0.001
Folate, nmol/L	13.62 (10.48–16.92)	11.50 (8.90–13.40)	<0.001
Homocysteine, μmol/L	11.34 (9.12–14.23)	15.96 (11.50–19.18)	<0.001
Clinical center			
Ha’rbin	35 (13.1)	24 (12.0)	<0.001
Shanghai	48 (17.9)	13 (6.5)	
Shenyang	44 (16.4)	34 (17.0)	
Beijing	67 (25.0)	47 (23.5)	
Xi’an	30 (11.2)	47 (23.5)	
Nanjing	44 (16.4)	35 (17.5)	
*MTHFR* C677T			
CC	64 (23.9)	50 (25.0)	0.880
CT	139 (51.9)	99 (49.5)	
TT	65 (24.3)	51 (25.5)	
*MTHFR* A1298C			
AA	190 (70.9)	136 (68.0)	0.260
AC	72 (26.9)	54 (27.0)	
CC	6 (2.2)	10 (5.0)	
*MTR* A2756G			
AA	222 (82.8)	160 (80.0)	0.367
AG	45 (16.8)	37 (18.5)	
GG	1 (0.4)	3 (1.5)	
*MTRR* A66G			
AA	77 (28.7)	76 (38.0)	0.089
AG	146 (54.5)	91 (45.5)	
GG	45 (16.8)	33 (16.5)	

### 3.2. Associations between Genotypes and Folate Level

The associations between the genotypes and the serum folate level are shown in Table 3. The patients with the *MTHFR* 677TT genotype had a lower serum folate level than the 677CC carriers (adjusted β (SE): −0.27 (0.01), *p* < 0.001). However, there was no significant difference in folate levels between the 677CT and 677CC genotypes. When the 677CC and 677CT genotypes were grouped together, we found that the 677TT carriers had a lower serum folate level than the 677CC + CT carriers (adjusted β (SE): −0.19 (0.02), *p* < 0.001). However, the patients with the *MTHFR* 1298AC + CC genotypes had a higher serum folate level than those with the wild-type genotype (adjusted β (SE): 0.10 (0.02), *p* = 0.025). Furthermore, the patients with *MTR* 2756AG + GG had a lower serum folate level than the 66AA carriers (adjusted β (SE): −0.12 (0.02), *p* = 0.005). There was no significant correlation between the *MTRR* A66G polymorphism and serum folate.

**Table 3:** Associations of gene polymorphisms with serum folate level.

Genotype	Folate ^1^	log(Folate) ^1^	Crude		Adjusted ^2^	
			β (SE)	*p*	β (SE)	*p*
*MTHFR* C677T						
CC (*n* = 114)	14.97 ± 6.23	1.14 ± 0.16	Reference		Reference	
CT (*n* = 238)	14.00 ± 6.16	1.11 ± 0.17	−0.09 (0.02)	0.098	−0.07 (0.02)	0.186
TT (*n* = 116)	11.85 ± 5.22	1.04 ± 0.16	−0.30 (0.01)	<0.001	−0.27 (0.01)	<0.001
CC + CT (*n* = 352)	14.32 ± 6.19	1.12 ± 0.17	Reference		Reference	
TT (*n* = 116)	11.85 ± 5.22	1.04 ± 0.16	−0.21 (0.02)	<0.001	−0.19 (0.02)	<0.001
*MTHFR* A1298C						
AA (*n* = 326)	13.35 ± 5.81	1.09 ± 0.16	Reference		Reference	
AC (*n* = 126)	14.62 ± 6.61	1.13 ± 0.17	0.10 (0.02)	0.039	0.10 (0.02)	0.026
CC (*n* = 16)	13.73 ± 6.15	1.10 ± 0.18	0.01 (0.02)	0.818	0.03 (0.02)	0.596
AA (*n* = 326)	13.35 ± 5.81	1.09 ± 0.16	Reference		Reference	
AC + CC (*n* = 142)	14.52 ± 6.54	1.13 ± 0.17	0.09 (0.02)	0.049	0.10 (0.02)	0.025
*MTR* A2756G						
AA (*n* = 382)	13.95 ± 6.26	1.11 ± 0.17	Reference		Reference	
AG + GG (*n* = 86)	12.61 ± 4.96	1.07 ± 0.17	−0.10 (0.02)	0.041	−0.12 (0.02)	0.006
*MTRR* A66G						
AA (*n* = 153)	13.57 ± 5.78	1.10 ± 0.17	Reference		Reference	
AG (*n* = 237)	13.83 ± 5.60	1.11 ± 0.16	0.04 (0.02)	0.480	0.02 (0.02)	0.731
GG (*n* = 78)	13.61 ± 7.76	1.09 ± 0.19	−0.04 (0.01)	0.600	−0.03 (0.01)	0.665
AA (*n* = 153)	13.57 ± 5.78	1.10 ± 0.17	Reference		Reference	
AG + GG (*n* = 315)	13.77 ± 6.19	1.10 ± 0.17	0.02 (0.02)	0.727	0.01 (0.02)	0.841

^1^ Data are presented as the mean ± standard deviation (nmol/L). ^2^ Adjusted for sex, age, clinical center, height, and weight.

The effects of the four genotypes on folate deficiency are shown in Table 4. Using the *MTHFR* 677CC + CT genotypes as references, the odds ratio of the 677TT carriers was 2.34 (95% CI 1.47–3.71, *p* < 0.001). Additionally, the patients with the *MTR* 2756AG + GG genotypes had a higher risk of folate deficiency than the 2756AA carriers (OR = 1.80, 95% CI 1.06–3.05, *p* = 0.030). However, the patients with *MTHFR* 1298AC + CC showed a lower risk of folate deficiency, although this finding did not reach significance. Additionally, we found no significant effect of the *MTRR* A66G polymorphism on folate deficiency.

**Table 4:** Effects of gene polymorphisms on folate deficiency.

Genotype	Low folate ^1^	High folate ^1^	OR (95%Cl)	*p* ^2^
*MTHFR* C677T				
CC	26 (19.8)	88 (26.1)	Reference	
CT	57 (43.5)	181 (53.7)	1.01 (0.58–1.74)	0.980
TT	48 (36.6)	68 (20.2)	2.35 (1.29–4.26)	0.005
CC + CT	83 (63.4)	269 (79.8)	Reference	
TT	48 (36.6)	68 (20.2)	2.34 (1.47–3.71)	<0.001
*MTHFR* A1298C				
AA	98 (74.8)	228 (67.7)	Reference	
AC	27 (20.6)	99 (29.4)	0.61 (0.37–1.01)	0.055
CC	6 (4.6)	10 (3.0)	1.18 (0.40–3.48)	0.770
AA	98 (74.8)	228 (67.7)	Reference	
AC + CC	33 (25.2)	109 (32.3)	0.67 (0.42–1.07)	0.095
*MTR* A2756G				
AA	100 (76.3)	282 (83.7)	Reference	
AG + GG	31 (23.7)	55 (16.3)	1.80 (1.06–3.05)	0.030
*MTRR* A66G				
AA	41 (31.3)	112 (33.2)	Reference	
AG	60 (45.8)	177 (52.5)	0.99 (0.61–1.60)	0.966
GG	30 (22.9)	48 (14.2)	1.65 (0.90–3.01)	0.105
AA	41 (31.3)	112 (33.2)	Reference	
AG + GG	90 (68.7)	225 (66.8)	1.14 (0.72–1.79)	0.579

^1^ Data are presented as the number of cases (percentage). Low folate: serum folate level < 10 nmol/L; high folate: serum folate level ≥ 10 nmol/L. ^2^ Adjusted for sex, age, clinical center, height, and weight.

### 3.3. Gene-Gene Interactions on Folate Deficiency

We next examined the joint effects of these four gene polymorphisms on deficiency (Table 5). None of the patients had the 677TT/1298AC + CC genotypes, consistent with some previous reports [21,22]. Haplotypes of these two mutations have been suggested to be in complete linkage disequilibrium (*p*-value < 0.0001) [26]. Compared with the 677CC + CT/1298AC + CC carriers, the patients with the 677TT/1298AA genotypes had higher odds of folate deficiency (OR = 2.45, 95% CI 1.41–4.28, *p* = 0.002). Furthermore, the patients with 677TT/2756AG + GG had a four-fold increased risk of folate deficiency compared with those carrying 677CC + CT/2756AA (OR = 4.13, 95% CI 1.68–10.13, *p* = 0.002), and the 677TT/66AG + GG carriers had higher odds of folate deficiency than the 677CC + CT/66AA carriers (OR = 2.50, 95% CI 1.33–4.67, *p* = 0.004). Additionally, the 1298AA/2756AG + GG and 2756AG + GG/66AG + GG carriers both had higher risks of folate deficiency compared with the reference group. We further performed the trend test to verify these findings and, except for the *MTHFR* A1298C/*MTRR* A66G combination, the other genotype combinations dramatically increased the folate deficiency risk.

We used the GMDR model to explore the effects of high-order gene-gene interactions on folate deficiency (Table 6). The model with *MTHFR* C677T, *MTHFR* A1298C, *MTR* A2756G, and *MTRR* A66G had the highest training balance accuracy (0.6357), a relatively high testing balance accuracy (0.5717), the maximum cross-validation consistency of 10/10, and a significant *p* value (*p* = 0.0107). Thus, this model is probably the best model for interpreting the effects of high-order gene-gene interactions on folate deficiency.

**Table 5:** Effects of gene-gene interactions on folate deficiency.

Genotype 1	Genotype 2	N_L_/N_H_^1^	OR (95%Cl)		*p* ^2^	Trend test	*p* ^2^
*MTHFR* C677T	*MTHFR* A1298C						
CC + CT	AC + CC	33/109	Reference			0.16 (0.02)	<0.001
CC + CT	AA	50/160	1.09 (0.65–1.82)		0.757		
TT	AC + CC	0/0	-		-		
TT	AA	48/68	2.45 (1.41–4.28)		0.002		
*MTHFR* C677T	*MTR* A2756G						
CC + CT	AA	65/225	Reference			0.19 (0.02)	<0.001
CC + CT	AG + GG	18/44	1.69 (0.88–3.23)		0.116		
TT	AA	35/57	2.22 (1.32–3.74)		0.003		
TT	AG + GG	13/11	4.13 (1.68–10.13)		0.002		
*MTHFR* C677T	*MTRR* A66G						
CC + CT	AA	29/91	Reference			0.15 (0.02)	0.001
CC + CT	AG + GG	54/178	1.02 (0.60–1.73)		0.954		
TT	AA	12/21	2.03 (0.84–4.87)		0.114		
TT	AG + GG	36/47	2.50 (1.33–4.67)		0.004		
*MTHFR* A1298C	*MTR* A2756G						
AC + CC	AA	26/92	Reference			0.11 (0.02)	0.019
AC + CC	AG + GG	7/17	2.07 (0.72–5.92)		0.177		
AA	AA	74/190	1.50 (0.89–2.54)		0.131		
AA	AG + GG	24/38	2.54 (1.26–5.15)		0.010		
*MTHFR* A1298C	*MTRR* A66G						
AC + CC	AA	13/38	Reference			0.08 (0.02)	0.088
AC + CC	AG + GG	20/71	0.87 (0.38–1.99)		0.743		
AA	AA	28/74	1.18 (0.54–2.60)		0.680		
AA	AG + GG	70/154	1.46 (0.72–2.98)		0.297		
*MTR* A2756G	*MTRR* A66G						
AA	AA	30/93	Reference			0.10 (0.02)	0.032
AA	AG + GG	70/189	1.16 (0.69–1.94)		0.570		
AG + GG	AA	11/19	1.73 (0.70–4.29)		0.240		
AG + GG	AG + GG	20/36	2.09 (1.01–4.29)		0.046		

^1^ N_L_: number of low folate patients (<10 nmol/L); N_H_: number of high folate patients (≥10 nmol/L); ^2^ Adjusted for sex, age, clinical center, height, and weight.

**Table 6:** GMDR models of effects of high-order interactions on folate deficiency.

Models ^1^	Training balance accuracy	Testing balance accuracy	Sign test (*p* value)	Cross-validation consistency
C677T	0.5842	0.5825	8 (0.0547)	10/10
C677T, A2756G	0.6047	0.5946	10 (0.0010)	10/10
C677T, A2756G, A66G	0.6236	0.5631	10 (0.0010)	6/10
C677T, A2756G, A1298C, A66G	0.6357	0.5717	9 (0.0107)	10/10

^1^ All models adjusted for sex, age, clinical center, height and weight.

**Table 7:** Cumulative effects of risk genotypes ^1^ on folate deficiency.

Number of risk genotypes	Low folate ^2^	High folate ^2^	OR (95%Cl)	*p* ^3^	*p*_trend_^3^
0	10 (7.6)	33 (9.8)	Reference		0.001
1	32 (24.4)	106 (31.5)	1.01 (0.44–2.32)	0.989	
2	40 (30.5)	132 (39.2)	1.17 (0.52–2.63)	0.710	
3	41 (31.3)	58 (17.2)	2.53 (1.10–5.85)	0.029	
4	8 (6.1)	8 (2.4)	3.77 (1.07–13.27)	0.039	
≥3	49 (37.4)	66 (19.6)	2.68 (1.18–6.09)	0.019	

^1^ Risk genotypes were defined as *MTHFR* 677TT, *MTHFR* 1298AA, *MTR* 2756AG + GG, and *MTRR* 66AG + GG. ^2^ Data are presented as the number of cases (percentage). Low folate: serum folate level <10 nmol/L; high folate: serum folate level ≥10 nmol/L. ^3^ Adjusted for sex, age, clinical center, height, and weight.

### 3.4. Cumulative Effects of Risk Genotypes on Folate Deficiency 

We defined the risk genotypes as 677TT, 1298AA, 2756AG + GG, and 66AG + GG. The cumulative effects of these four polymorphisms on folate deficiency are shown in Table 7. Due to the numbers of patients with all these four risk genotypes being relatively low, we combined the patients carrying three or four risk genotypes for evaluation. Compared with the null risk genotype carriers, the patients carrying three risk genotypes had a higher folate deficiency risk, with an odds ratio of 2.53 (95% CI 1.10–5.85; *p* = 0.029). In addition, the patients with four risk genotypes had a nearly four-fold increased risk of folate deficiency (OR = 3.77, 95% CI 1.07–13.27; *p* = 0.039). When we combined the patients with three or four risk genotypes, the increase in the folate deficiency risk remained significant (OR = 2.68, 95% CI 1.18–6.09; *p* = 0.019). Furthermore, the trend test showed a dramatic increase in folate deficiency with an increase in the number of risk genotypes (*p*_trend_ = 0.001). Plausibly, these results further support the conclusion drawn from the GMDR results that potential interactions among these gene polymorphisms may affect the incidence of folate deficiency.

## 4. Discussion

In the present study, the *MTHFR* C677T and *MTR* A2756G polymorphisms each independently reduced the serum folate level and increased the folate deficiency risk. In addition, compared with the wild-type of *MTHFR* A1298C genotype, patients carried the mutant C allele showed higher folate level. We defined the risk genotypes as *MTHFR* 677TT, *MTHFR* 1298AA, *MTR* 2756AG + GG, and *MTRR* 66AG + GG. Assessment of gene-gene interactions using the ULR and GMDR models revealed significant effects of interactions of these four risk genotypes on folate deficiency.

Folate is a crucial factor in cell division and cell maintenance and also plays an important role in regulating epigenetic gene expression [12]. A recent study has shown that three forms of folate supplementation (natural folate-rich foods, folic acid and 5-MTHF) have similar effects on lowering plasma homocysteine [8]. Pravenec *et al.* have indicated that a reduction in the folate level aggravates evidence of oxidative tissue damage and insulin resistance, and elevates blood pressure in spontaneously hypertensive rats [27]. We found that a low folate level resulted in a significant increase in the plasma homocysteine concentration (*p* < 0.001, data not show) and this negative correlation has been widely confirmed [10,23,25,28]. Furthermore, several studies showed that folate supplementation reduced the plasma homocysteine level, urinary 8-iso-prostaglandin F_2α_ and 11-dehydro-thromboxane B_2_ excretion, and increased serum folate level especially in hyperhomocysteinemic carriers [29,30]. These results showed the beneficial of folate supplementation on oxidative stress and platelet activation. In addition, a prospective study has shown that folate deficiency is independently predictive of a 53% increased risk of coronary heart disease mortality in older adults [31]. In an Indian cohort, the *MTHFR* 677T allele and folate deficiency independently increased neonatal hyperbilirubinemia risk by approximately four-fold and three-fold, respectively [32]. These findings indicate that folate deficiency may independently increase the risk of CVDs.

According to the threshold of 10 nmol/L, approximately 28% (131) of the Chinese hypertension patients in our study were folate deficient. Toprak *et al.* reported only 201 (1.1%) subjects with a folate level of ≤5 nmol/L and 640 (4.7%) with a level of ≤6.8 nmol/L out of a total of 17,713 participants [33], suggesting that use of a higher folate cutoff value may improve sensitivity for detecting a deficiency. The relationship between folate and homocysteine may differ depending on whether the folate level is low or high. Selhub *et al.* have reported that the serum homocysteine concentration increases as the serum folate concentration decreases; however, at a high folate level, this correlation may disappear [25]. In addition, the two-phase regression model suggests that the threshold folate level is approximately 10 nmol/L, which is the level at which the homocysteine concentration approaches flat [25]. Therefore, in this study, we defined folate deficiency as a serum folate level of <10 nmol/L.

We observed that the patients with *MTHFR* 677TT had a lower serum folate level (*p* < 0.001, Table 3) and a higher odds of folate deficiency (*p* < 0.001, Table 4) than the 677CC + CT carriers. This negative correlation between *MTHFR* C677T polymorphisms and the folate level has been widely reported [7,10,11,19,34]. A previous study has reported that *MTHFR* 677TT may cause an approximately 70% decrease and 677CT results in a 35% decrease in the mean MTHFR activity [35]. The *MTHFR* C677T mutation is located in the catalytic domain of the enzyme [35,36] and causes an alanine to valine substitution at position 222, resulting in a thermolabile enzyme [22,35]. The homozygous *MTHFR* C677T mutation decreases enzymatic activity and causes a lower rate of reduction of 5, 10-methylene-THF to 5-methyl-THF, resulting in increased availability of 5, 10-methylene-THF for oxidation to the formylated folate forms and accumulation in red blood cells (RBCs) [37]. However, no formylated folates have been found in RBCs of 677CC genotype carriers. Furthermore, because mature RBCs have almost no ability to transport folate, accumulation of formylated-THF RBCs may lead to lower proportions of 5, 10-methylene-THF and 5-methyl-THF in the serum, which could explain the decreased serum folate level observed in individuals with the 677TT genotype. However, Waskiewicz *et al.* failed to find this association in either adult Polish men or women [38]. Moreover, a low folate level and *MTHFR* 677TT may have a synergistic effect on elevating the plasma homocysteine concentration [10,11]. *MTHFR* A1298C is located in the regulatory domain named NADPH and S-adenosylmethionine binding site [36]. The A1298C mutation does not result in synthesis of a thermolabile protein [22]. Thus, this mutation may not have a significant effect on the serum folate level (AA *vs.* AC + CC, *p* = 0.684) [7]. Further, Yakub *et al.* have not found an effect of *MTHFR* A1298C on the serum folate level [10]. Our results showed that heterozygous and homozygous A1298C mutations resulted in a higher folate level compared with that resulting from the wild-type genotype (adjusted *p* = 0.025, Table 3). Furthermore, *MTHFR* 1298AC + CC may be a protective factor that reduces the risk of folate deficiency, although this finding was not significant (Table 4). Similar to our results, Biselli *et al.* have found that the mean plasma folate concentration is significantly higher in carriers of the altered allele (1298AC and 1298CC) compared with carriers of 1298AA in coronary artery disease patients [18]. One possible reason for this finding is that S-adenosylmethionine is an allosteric inhibitor of MTHFR [36]. Structural prediction revealed that the S-adenosylmethionine binding site in the mutated structure is distorted compared with that in the normal structure [36] and therefore, it may reduce the inhibitory effect of the enzyme. However, in contrast with the results of our study, subjects carrying the 1298CC genotype have been demonstrated to have a lower serum folate level compared with the levels in 1298AC and 1298CC carriers in a study of male clear cell renal cell carcinoma patients and control individuals [19]. Therefore, the potential impact of *MTHFR* A1298C on the serum folate level needs more investigation.

The physiologically active coenzyme form of folate is tetrahydrofolate (THF). *In vivo*, THF is converted into 5, 10-methylene-THF with the aid of vitamin B_6_ and then into 5-methyl-THF. The conversion of 5, 10-methylene-THF into 5-methyl-THF is physiologically irreversible [25]. Therefore, reduced MTR activity may decrease the rate of conversion of 5-methyl-THF to THF, then results in physiological folate deficiency. *MTR* A2756G mutation located at position 919 of the protein results in substitution of glycine for aspartic acid [39]. It is located in a domain of the protein that interacts with S-adenosylmethionine and auxiliary proteins that are required for the reductive methylation and reactivation of the vitamin B_12_ cofactor, which can be inactivated by oxidation during catalysis [17,40]. Therefore, this mutation might impair the binding of SAM and/or auxiliary proteins [40] and reduce catalytic efficiency. In this study, we found that the *MTR* 2756AG + GG genotypes resulted in not only a decreased serum folate level (*p* = 0.006, Table 3) but also an increased folate deficiency risk compared with the wild-type genotype (*p* = 0.030, Table 4). In contrast, studies of the Brazilian [7], Pakistani [10], and Jamaican [20] populations have failed to demonstrate a correlation between the *MTR* A2756G polymorphism and folate level. However, serum folate and the *MTHFR* 677CC+CT and *MTR* 2756AA genotypes have been shown to have significant interactions with total homocysteine in pregnant women [7]. Furthermore, in individuals with low intake of folate, vitamin B_6_, and vitamin B_12_, the *MTHFR* 677T and *MTR* 2756G alleles have been shown to result in a high risk of breast cancer [41].

A common *MTRR* polymorphism is the substitution of A for G at nucleotide 66, which results in the substitution of isoleucine by methionine. This mutation is located in the putative flavin mononucleotide-binding domain of the MTRR enzyme, which interacts with MTR [17] and, thus, disrupts the binding of MTRR to the MTR-cobalamin-complex, decreasing the rate of homocysteine remethylation [39]. Our results showed that *MTRR* 66AG + GG may not affect the serum folate level or the incidence of folate deficiency (Table 3 and Table 4). Similarly, Feix *et al.* have reported that the *MTRR* A66G polymorphism has no effects on the total homocysteine, folate or vitamin B_12_ concentrations [42]. Although the *MTRR* A66G polymorphism has no significant influence on the serum folate level, the combination of *MTHFR* C677T and *MTRR* A66G have significant interactive effects on the total homocysteine and serum folate concentrations [7].

Based on these findings, we hypothesized that multiple genetic defects will aggravate folate deficiency. Several studies showed the combination effect of *MTHFR* C677T and A1298C decreased serum folate level [7,19,22]. MTHFR is crucial for maintaining an adequate methionine pool and for ensuring that the homocysteine concentration does not reach a toxic level [43]. The homozygous *MTHFR* C677T mutation causes a decrease in the conversion rate of 5, 10-methylene-THF to 5-methyl-THF resulting in a reduction in the 5-methyl-THF level [35]. Subsequently, the supply of methyl groups is diminished, affecting the synthesis of methionine from homocysteine. *MTR* 2756G allele affects the binding of accessory proteins involved in cofactor reduction [44], potentially resulting in reductions in the synthesis rates of 5-methyl-THF to THF and homocysteine to methionine. A low THF level also affects folate circulation. Furthermore, MTRR restores the activity of MTR [17]. Thus, a mutation in MTRR may result in a decreased recovery rate of the oxidant cobalamin, indirectly affecting the serum folate level. As such, the deleterious effects of homocysteine metabolism gene polymorphisms on serum folate level are biologically plausible. In Brazilian children, the folate level has been shown to be significantly decreased in subjects with the 677CC/1298AA/66AA genotypes compared with those carrying 677CC/1298AA/66AG (*p* = 0.03), 677CC/1298AC/66AG (*p* = 0.003) and 677CT/1298AA/66AG (*p* = 0.02) [21]. In our study, the assessment of high-order gene-gene interactions with our GMDR model revealed that all four gene polymorphisms interactively influenced the prevalence of folate deficiency (Table 6). The results from the evaluation of cumulative effects verified these results (Table 7).

To the best of our knowledge, we are the first to demonstrate that the *MTHFR* C677T, *MTHFR* A1298C, *MTR* A2756G, and *MTRR* A66G gene polymorphisms have significant interactive effects on the risk of folate deficiency in Chinese hypertensive patients. A limitation of our study is that the sample size was relatively small. Therefore, we believe that future studies with large samples could be performed to validate our results in a more expansive population.

## 5. Conclusions

Folate deficiency is a risk factor for cardiovascular disease that is modified by several gene polymorphisms. This study showed that *MTHFR* 677TT, *MTHFR* 1298AA, and *MTR* 2756AG + GG are independently correlated with a high risk of folate deficiency. Furthermore, we have demonstrated that not only pairwise gene-gene interactions but also higher-order interactions of these gene polymorphisms (*MTHFR* C677T, *MTHFR* A1298C, *MTR* A2756G, and *MTRR* A66G) more strongly influence the incidence of folate deficiency. We suggest that individuals who carry those risk genotypes (especially for multiple risk genotypes carriers) should be monitored for their folate circulating levels, and have folate supplementation in case of deficiency. Whether this intervention may translate into a meaningful reduction of cardiovascular events will be hopefully unraveled in the next years.
